# A Predictive Growth Model for Pro-technological and Probiotic *Lacticaseibacillus paracasei* Strains Fermenting White Cabbage

**DOI:** 10.3389/fmicb.2022.907393

**Published:** 2022-06-06

**Authors:** Mariaelena Di Biase, Yvan Le Marc, Anna Rita Bavaro, Palmira De Bellis, Stella Lisa Lonigro, Paola Lavermicocca, Florence Postollec, Francesca Valerio

**Affiliations:** ^1^Institute of Sciences of Food Production, National Research Council of Italy, Bari, Italy; ^2^ADRIA Food Technology Institute, UMT ACTIA 19.03 ALTER’iX, Creac’h Gwen, Quimper Cedex, France

**Keywords:** *Lacticaseibacillus paracasei*, growth models, fermented cabbage, predictive modeling, probiotic foods

## Abstract

Bacterial strains belonging to *Lacticaseibacillus paracasei* species are generally used as starters in food fermentations and/or as probiotics. In the current study, the growth cardinal parameters of four *L. paracasei* strains (IMPC2.1, IMPC4.1, P40 and P101), isolated from table olives or human source, were determined. Strains were grown in liquid medium and incubated at several temperatures (10 values from 5.5°C–40°C) and pH (15 values from 3.2 to 9.1) along the growth range. The cardinal temperature model was used to describe temperature effects on the maximum specific growth rate of *L. paracasei* whereas new equations were developed for the effect of pH. The estimated *T_min_* values ranged between −0.97°C and 1.95°C and were lower than 0°C for strains IMPC4.1 and P101. Strain P40 was able to grow in the most restricted range of temperature (from 1.95°C to 37.46°C), while strain IMPC4.1 was estimated to survive at extreme conditions showing the lowest *pH_min_*. Maximum specific growth rates of *L. paracasei* IMPC2.1 in white cabbage (*Brassica oleracea* var. *capitata*) were used to calculate the correction factor (*C_f_*) defined as the bias between the bacterial maximum specific growth rate in broth and in the food matrix. A simple bi-linear model was also developed for the effect of temperature on the maximum population density reached in white cabbage. This information was further used to simulate the growth of *L. paracasei* strains in cabbage and predict the time to reach the targeted probiotic level (7 log_10_ CFU/g) using *in silico* simulations. This study demonstrates the potential of the predictive microbiology to predict the growth of beneficial and pro-technological strains in foods in order to optimize the fermentative process.

## Introduction

Since the 1990s, predictive microbiology has been mainly used to identify microbial hazards and to predict their bacterial population development or destruction to ensure food safety. These approaches have been applied to pathogenic strains, e.g., *Salmonella*, *Listeria monocytogenes*, *Escherichia coli* (e.g., Couvert et al., 2010; Koutsoumanis et al., 2016) and strains involved in spoilage (e.g., Valerio et al., 2015a). For this purpose, several microbial databases, model repositories and decision tools (e.g., Sym’Previus)^1^ have been created from microbial data sets, relevant to growth and inactivation kinetic data. Based on mathematical equations describing and predicting bacterial behavior, predictive microbiology allows to evaluate the impact of intrinsic and extrinsic parameters (pH; temperature, T; water activity, a_w_) on bacterial growth and destruction. Coupling mathematical modeling and challenge test data enables to optimize food processes and evaluate shelf-life during food storage or distribution. To our knowledge, few studies have been performed until now on the application of predictive microbiology to technological strains for the optimization of fermentation process (Dorota et al., 2014; Nikmaram et al., 2016).

Beneficial microorganisms as the lactic acid bacteria (LABs) are widely used in food manufacturing for their technological and potential functional properties (Axelsson and Ahrné, 2000). LABs are generally used as starter cultures to pilot food fermentations (Leroy and De Vuyst, 2004), to confer functional properties to foods when used as probiotics (Lavermicocca et al., 2005; Valerio et al., 2006; De Bellis et al., 2010; Sarvan et al., 2013), to prolong food shelf-life when applied as bioprotective cultures (Lavermicocca et al., 2000, 2003; Ström et al., 2005; Dal Bello et al., 2007; Gerez et al., 2009; Valerio et al., 2013; Di Biase et al., 2014). Among LAB strains, the probiotic and technological properties of some *Lacticaseibacillus paracasei* strains have been demonstrated (Komatsuzaki et al., 2005; Verdenelli et al., 2009; Gudiña et al., 2010; Chuang et al., 2011), as for strain *L. paracasei* IMPC4.1 which showed interesting bio-preservative and anti-inflammatory abilities (D’Arienzo et al., 2011; Sisto et al., 2016; Danza et al., 2018). Moreover, strain IMPC2.1 was proven to survive the simulated and *in vivo* gastrointestinal digestion, to exert immunomodulatory, anti-proliferative and pro-apoptotic effects (Lavermicocca et al., 2005; D’Arienzo et al., 2011; Orlando et al., 2012; Sisto et al., 2016; Danza et al., 2018) and to be suitable for industrial processing to produce probiotic ready-to-eat foods such as olives, white cabbage, artichokes, fillet fish and dehydrated apple slices (De Bellis et al., 2010; Valerio et al., 2010, 2011, 2015b, 2020; Sarvan et al., 2013).

In particular, some of these ready-to-eat foods enriched with the probiotic *L. paracasei* IMPC2.1 have been tested in human feeding studies and showed promising and successful health benefits (Lavermicocca et al., 2005; Valerio et al., 2010, 2011, 2015b; Riezzo et al., 2012). In these trials, the efficacy of the strain to reach in adequate amounts the gut and to act as a probiotic, has been demonstrated. The food portions contained about 9 log_10_ CFU of cells corresponding to 7 log_10_ CFU/g which was further used as a targeted level for technological flora fermentation using predictive microbiology approaches. Moreover, Valerio et al. (2010) and Riezzo et al. (2012) registered the health benefits on constipated subjects after consuming *L. paracasei* IMPC2.1 enriched ready-to-eat artichokes, previously authorized by the Italian Ministry of Health for the market. In fact, as established by FAO (FAO/WHO, 2002), reported in Hill et al. (2014) and evidenced for strain IMPC2.1 by Valerio et al. (2010) and Riezzo et al. (2012), a probiotic food should contain at least 10^9^ viable cells per portion to exert its beneficial effects, but the processing conditions, including extrinsic (temperature) and intrinsic characteristics of the food matrix (a_w_, pH), could limit the strain ability to reach this cell concentration in a fermentation process. It is known that exposure to continuous changing environment which occurs during food fermentation induces adaptive behavior in bacterial metabolism (e.g., gene expression, physiological and molecular mechanisms; Papadimitriou et al., 2016).

The beneficial effects of a probiotic strain could also be associated with the functional benefits of some food components, as the prebiotic inulin contained in the artichokes or other bioactive molecules. The carrier food and particularly plant-based matrices could contain phytochemicals and can be successfully exploited for carrying probiotic populations (De Bellis et al., 2021). Vegetables belonging to *Brassicaceae* family are rich in polyphenols and carotenoids, but the glucosinolate fraction represents its most important dietary group of bioactive molecules. The preservation of these antioxidant and health-promoting compounds is highly dependent on the processing they underwent: an appropriate heat treatment inactivating the myrosinase, followed by a piloted fermentation can contribute to save glucosinolates in fermented cabbage as previously demonstrated using the *L. paracasei* IMPC2.1 as a starter to ferment white cabbage (*Brassica oleracea* var. *capitata*; Sarvan et al., 2013). As a result, this vegetable matrix, containing glucosinolates (GSs), resulted to be a suitable substrate for *L. paracasei* growth and for the transformation in a functional vegetable product containing both glucosinolates and an adequate probiotic level (about 7 log_10_ CFU/g).

For these reasons, predictive microbiology could significantly contribute to optimize industrial processes which foresees the application of pro-technological strains, through the use of validated mathematical models which obtained a preliminary consensus by the International Organization for Standardization (ISO 20976-1:2019; International Organization for Standardization, 2019).

In that context, the aim of the current study was to develop a predictive growth model for *L. paracasei* spp. in white cabbage in order to select the fermentation conditions allowing to reach a targeted level of cells of 7 log_10_ CFU/g associated with a beneficial effect. In order to take into account biological variability, the determination of growth ability was performed for four pro-technological and/or probiotic *L. paracasei* strains. Growth ability, also called cardinal values, was determined for both temperature and pH in culture medium. To account for food matrix, challenge tests were performed in white cabbage to determine the growth rate and calculate the food correction factor (Buss da Silva et al., 2017; Ellouze et al., 2021), noted *C_f_* in this study. This factor enables to correlate the growth rate obtained in broth and in a food matrix for further *in silico* simulations.

## Materials and Methods

### Bacterial Strains

Four *Lacticaseibacillus paracasei* strains, recently re-classified by Zheng et al. (2020) and thereafter named *L. paracasei*, were used in the study. Two strains, *L. paracasei* IMPC2.1 (LMG P-22043; ITEM 17146) and IMPC4.1 (LMG S-27068; ITEM 15479), isolated from human source belonging to the ITEM Culture Collection of the Institute of Sciences of Food Production, National Research Council (ISPA-CNR)^2^ and deposited in the Belgian Coordinated Collections of Microorganisms (BCCM/LGM, Gent, Belgium), were used as strains representative of a probiotic species also applied in the production of fermented foods (Lavermicocca et al., 2005, 2008; Valerio et al., 2006). *L. paracasei* P40 and P101 were previously isolated from table olives (De Bellis et al., 2010). For long-term storage, stock cultures were prepared by mixing 8 ml of a culture in de Man Rogosa Sharpe (MRS) broth (Oxoid Ltd., Basingstoke, United States) with 2 ml of Bacto glycerol (Difco, Detroit, MI., United States) and freezing 1 ml portions of this mixture at −80°C. To obtain fresh cultures, the strains were subcultured twice (1% vol/vol) in MRS broth for 24 h before use.

### Genotypic Identification and Characterization of Bacterial Strains

Genomic DNA was extracted from cultures grown in MRS broth (Oxoid, United Kingdom) at 37°C for 24 h, using a CloneSaver Card Kit (Whatman, Maidstone, United Kingdom). The identification of strains, previously carried out by sequencing of the 16S rRNA gene (Sisto et al., 2009; De Bellis et al., 2010), was confirmed by a multiplex-PCR based on species-specific primers designed on *tuf* gene (Ventura et al., 2003). The strains were also analyzed by rep-PCR using the primer pair REP-1R-Dt/REP-2R-Dt, REP 1R-I/REP 2-I, the primer BOX A1R and the primer (GTG)_5_ as previously described (Valerio et al., 2012). Moreover, the *Lactiplantibacillus plantarum* ITM21B (LMG P-2203) was used as an internal control in different PCR reactions and chip runs, and included in the analysis and used as outgroup in the characterization. The rep-PCR profiles were analyzed using the DNA7500 LabChip Kit and the Bioanalyzer2100 platform (Agilent Technologies, Waldbronn, Germany). DNA 7500 ladder was used as size standard and as a normalization reference, in addition to Lower (50 bp) and Upper (10.380 bp) markers added to each DNA sample on chip. The reproducibility of the fingerprints was verified by repeating the analysis twice. Dice similarity coefficient with 1.0% band position tolerance and UPGMA algorithm were used for cluster analysis by BioNumerics v.5.0 software (Applied Maths, Inc., Austin., TX, United States).

### Experimental Conditions

#### Growth of *Lacticaseibacillus paracasei* Strains in Broth

Growth parameters for temperature and pH (e.g., minimal, optimal and maximal values of temperature and pH for growth) were determined from experiments performed in broth. Each factor was tested in a mono-factorial design. Growth kinetics were performed in 0.22 μm filtered modified de Man Rogosa and Sharpe (mMRS) broth lacking acetic acid and citrate (pH 6.2), according to Cuppers and Smelt (1993). The pH value of medium was adjusted using 5 N HCl or 5 N NaOH to mMRS and recorded by pH meter (Beckman Coulter, model 340, supplied with a glass electrode Beckman Coulter, Brea, CA). For all strains, the maximum specific growth rate (*μ_max_*) was determined for about 10 temperatures (from 5.5 to 40°C) and 15 pH values (from 3.2 to 9.1). The pH experiments were conducted at 30°C. For studied temperatures range from 17°C to 40°C, the growth of strains was automatically monitored by a Bioscreen C (Labsystems, Helsinki, Finland) using the turbidimetry method (Membré et al., 2002) according to the dilution methods reported by Cuppers and Smelt (1993). Twenty controls (two technical replicates per experimental condition) were performed for each plate reader. Optical density (OD) measurements at 600 nm were taken at 27-min intervals. For each condition two technical replicates and two biological replicates were included. The recorded temperature of Bioscreen C chamber was stable (±0.1°C) at each set temperature. When growth kinetics could not be automatically recorded by the Bioscreen C, they were determined manually after static incubation in mMRS at tested temperatures (5.5°C and 11°C) using the same culture procedures of the inoculum preparation. Briefly, 50 ml of mMRS broth was inoculated with an initial cell concentration of 10^3^ CFU/ml for each experimental condition. The recorded temperature of incubators, monitored by using electronic HOBO Temp/RH data logger (Onset Corp., MA, United States), was stable (±0.1°C) at each set temperature. At appropriate time intervals, aliquots of the cultures were decimally diluted in sterile NaCl (0.85% w/v) + Tween 80 (0.025%) and 100 μl of each dilution was spread on MRS agar plates incubated for 48 h at 37°C.

#### Growth of *Lacticaseibacillus paracasei* IMPC2.1 in White Cabbage

Strain *L. paracasei* IMPC2.1 was selected for further experiments in mild processed white cabbage due to its probiotic and technological properties. This strain was reported as able to grow in cabbage reaching counts of about 8 log_10_ CFU/g after 51 h of fermentation in 4% (w/vol) NaCl brine (Sarvan et al., 2013).

Experimental protocols in white cabbage and data analysis were performed according to standardized and reported methods in order to ensure reliable simulations of growth in food (Pinon et al., 2004; Leporq et al., 2005). In the current study, 4 isothermal conditions were tested (at temperatures ranging from 15°C to 35°C, Table 1). Note that additional data from growth curves in white cabbage (25°C ± 0.2°C, pH 6.70 ± 0.2, N_0_ 3.54 ± 0.77) not included in Sarvan et al. (2013) were also compared with validation purposes and in particular, at each sampling time, aliquots of brine and cabbage, liquid and solid phases of the fermented product, were analyzed.

**Table 1:** Fermentation starting conditions (1–5) and growth kinetic parameters relevant to the growth of *Lacticaseibacillus paracasei* IMPC2.1 in blanched white cabbage.

Fermentation starting conditions^a^				Growth kinetic parameters^b^				
Condition	log_10_(*N_0_*; log_10_ CFU/g)	T (°C) ± 0.1	pH ± SD	*lag time* (h)	log_10_(*N_max_*; log_10_ CFU/g)	*K* (h_0_)	*μ_max_* (h^−1^)	*μ_max pred_* (h^−1^)
1 Sarvan et al., 2013	3.72 ± 0.3	25.0	6.04 ± 0.16	10.8	8.38	2.7756	0.257	0.287
2	4.50 ± 0.09	20.0	6.82 ± 0.014	11.1	7.46	2.1756	0.196	0.184
3	3.42 ± 0.15	25.0	5.86 ± 0.007	4.1	7.95	0.9307	0.227	0.29
4	2.15 ± 0.21	35.0	5.85 ± 0.028	0	7.9	-	0.47	0.36
5	4.36 ± 0.13	15.0	5.89 ± 0.007	0	6.78	-	0.101	0.117

*Each condition was obtained modifying the inoculum load, pH, and temperature values*.

a *log_10_(N_0_): inoculum load of strain IMPC2.1; T(°C): isothermal fermentation temperature; pH value at the beginning of fermentation*.

b *lag time*: *initial phase of growth; log_10_(*N_max_*): maximum population density; *K*: lag time assumed to be independent on the experimental conditions but dependent on cell history and food matrix; *μ*_*max*:_ maximum growth rate of strain IMPC2.1 in cabbage; *μ_max pred_*: maximum growth rate of strain IMPC2.1 predicted in cabbage considering the correction factor *C_f_.* The growth kinetic parameters were calculated as specified in sections Temperature and pH Models and Food Correction Factor, Lag Time and Effect of Temperature on the Maximum Population*.

Cabbage samples (*B. oleracea* var. *capitata*), purchased from a local market, were prepared and artificially inoculated with *L. paracasei* IMPC2.1, following the procedure reported by Sarvan et al. (2013) which included a blanching treatment (100°C for 5 min) to inactivate the thermolabile endogenous myrosinase, thus obtaining a higher GSs content compared to traditionally fermented cabbage. Briefly, aliquots of 50 g of blanched cabbage stripes and 100 ml of 4% (w/vol) NaCl brine were placed into 200-ml plastic sterile stomacher bags. The *L. paracasei* IMPC2.1 strain was inoculated in each bag according to the different fermentation conditions (2–5, Table 1 and additional growth curves as detailed above in this paragraph). All bags were placed at the selected temperatures which were monitored as described in section Growth of *Lacticaseibacillus paracasei* Strains in Broth. At different time intervals, samples were collected for microbiological analysis and determination of pH. Each experimental condition was performed in duplicate.

The pH was recorded by using a glass Double Pore Slim electrode (Hamilton, Bonaduz, Switzerland) connected to a portable pH meter (type110, Eutech Instruments, Singapore).

At each sampling time, dripped-off aliquots of cabbage were analyzed. Cabbage (25 g) was taken and homogenized in 225 g of sterile Buffered Peptone Water (Difco Laboratories, Detroit, MI, United States) for 2 min in a Stomacher (Seward, London, United Kingdom); decimal dilutions (100 μl) were plated in duplicate on MRS agar and incubated anaerobically at 37°C for 48 h. To monitor the presence of *L. paracasei* IMPC2.1 during the experiments, at each sampling time, the 20% of total colonies randomly picked from countable MRS agar plates, were isolated and checked for purity. Bacterial DNA from presumptive LABs was extracted from overnight cultures grown in MRS broth at 37°C and analyzed by rep-PCR using the primer pair REP-1R-Dt/REP-2R-Dt, as described in section Genotypic Identification and Characterization of Bacterial Strains. The identification of *L. paracasei* IMPC2.1 was based on the comparison of the specific pattern obtained from a pure culture of the strain with the rep-PCR profile of each isolate from cabbage (De Bellis et al., 2010).

### Mathematical Modeling

#### Determination of the Maximum Specific Growth Rates

For the Bioscreen experiments, the maximum specific growth rate (*μ_max_*) values were directly derived from the slope of the linear relation between time to detection (TTD) and the logarithm of inoculum size (Cuppers and Smelt, 1993). The TTD was defined as the time at which a certain well reaches a specific OD_600_ value of 0.15. In the manual experiments (either in broth or in white cabbage), the maximum specific growth rate (*μ_max_*) was calculated by fitting the growth curves to the logistic model with delay and rupture (Rosso et al., 1996). Secondary model fittings were performed with nonlinear fitting module (NLINFIT, MATLAB, R2019b, the MathWorks, United States). Before fitting, an usual square root transformation was performed to homogenize the variance of the maximum specific growth rate. Simulations of bacterial growth were performed using an in-house program developed in the MATLAB language.

#### Temperature and pH Models

A multiplicative model (Eq. (1)) was used to relate *μ_max_* to temperature and pH:


(1) $$\mu_{max}\left(T,\;pH\right)=\mu_{\mathrm{opt},MRS}\;\tau \left(T\right)\gamma \left(pH\right)$$

where *τ (T)*, *γ (pH)* are the normalized effects of temperature and pH, respectively, and *μ_opt,MRS_* is the optimum maximum specific growth rate (*μ_max_*) in modified MRS.

For the effect of temperature, the Cardinal Temperature Model with Inflection (CTMI, Rosso et al., 1993) was used:


(2) $$\tau \left(T\right)=\frac{\left(T-T_{\text{max}}\right){\left(T-T_{\text{min}}\right)}^{2}}{\;\left(T-T_{\text{min}}\right)\left(T_{\mathrm{opt}}-T_{\text{min}}\right)\left(T-T_{\mathrm{opt}}\right)-\left(T_{\mathrm{opt}}-T_{\text{max}}\right)\left(T_{\mathrm{opt}}+T_{\text{min}}-2T\right)}$$

where *T_min_*, *T_opt_* and *T_max_* are the minimum, optimum and maximum temperature for growth.

Most pH models assume a close-to-linear evolution of the growth rate as a function of suboptimal (and supraoptimal) pH. As this was not in accordance with our experimental observations, we have used the following term, based on Presser et al. (1997) and Presser (2001):


(3) $$\gamma \left(pH\right)=\frac{\left(1-10^{\left(pH_{\text{min}}-pH\right)}\right)\left(1-10^{Q\left(pH-pH_{\text{max}}\right)}\right)}{\left(1-10^{\left(pH_{\text{min}}-pH_{ref}\right)}\right)\left(1-10^{Q\left(pH_{ref}-pH_{\text{max}}\right)}\right)}$$

where *pH_min_* and *pH_max_* are the minimum, maximum pH for growth, respectively, and *Q* is a shape parameter. The parameter *pH_ref_* was set to 6.2 (i.e., the pH of the mMRS medium). Equation (3) describes a linear evolution of *μ_max_* as a function of proton concentration in the suboptimal pH range (Presser et al., 1997). The parameter *Q* reflects the shape of the relationship pH-*μ_max_* in the supraoptimal range (Presser, 2001). A value of *Q* equal to 1 would correspond to a linear evolution of *μ_max_* as a function of hydroxide ion concentration. Note also that *γ (pH)* is equal to 1 at the pH of the mMRS medium.

#### Food Correction Factor, Lag Time, and Effect of Temperature on the Maximum Population

The food correction factor (*C_f_*), the lag time model and the model for the effect of temperature on the maximum population were established using the experiments carried out in fermenting white cabbage.

To predict the maximum specific growth rate of *L. paracasei* in fermenting white cabbage, we calculated a “correction factor” *C_f_.* The correction factor compares the ability of a certain food matrix to support the growth of the studied strain in comparison with the growth obtained in culture medium (Buss da Silva et al., 2017; Ellouze et al., 2021). A correction factor closes to 1 indicates similar growth of the studied strain in the food product and in the culture medium. The *μ_max_* value predicted (*μ_max pred_*) in white cabbage is simply the predicted value in mMRS multiplied by the correction factor:


(4) $$\mu_{\max\mathrm{pred}}\left(T,pH\right)=C_{f}\;\mu_{\mathrm{opt},MRS}\;\tau \left(T\right)\gamma \left(pH\right)$$

The product *C_f_* *μ_opt,MRS_ represents here the optimum maximum growth rate in white cabbage. The *C_f_* value for strain IMPC2.1 was estimated as the value minimizing the sum square difference between the square root of predicted and observed *μ_max_* values in fermented cabbage at 15°C, 20°C, 25°C and 35°C for all conditions reported in Table 1.

The lag time was calculated through the parameter *K* (also called the “work to be done” h_0_, Robinson et al., 1998) which was assumed to be independent on the experimental conditions (e.g., Baranyi and Roberts, 1994; Rosso, 1995) but dependent on cell history and food matrix.


(5) $$K=\mu_{\text{max}}*\mathrm{lag}\;\mathrm{time}$$

The value for *K* in white cabbage was calculated as the geometric mean of the individual *K_i_* values calculated for the different experiments in food (Table 1).

The experimental data suggested that the maximum population density (*N_max_*) in white cabbage varies according to the fermentation temperature. To describe this relationship, we used the following equation:


(6) $$\left\{ \begin{array}{c} \log_{10}\left(N_{\text{max}}\right)=a_{0}+a_{1}\;T\;\mathrm{if}\;T<T_{c}\\ \;\log_{10}\left(N_{\text{max}}\right)=a_{0}+a_{1}\;T_{c}\;\mathrm{if}\;T\geq T_{c} \end{array}\right.$$

where *a_0_*, *a_1_* and *T_c_* are the model parameters. *T_c_* represents the temperature above which the *N_max_* is constant.

#### Simulation of the Growth of *Lacticaseibacillus paracasei* in White Cabbage

The growth of *L. paracasei* was simulated during fermentation of white cabbage for different conditions. The biological variability was taken into account by using probability distributions for the strain-dependent parameters (e.g., *μ_opt,MRS_*, cardinal temperatures, minimum pH for growth). As in Couvert et al. (2010), normal distributions defined by the calculated means and standard deviations of the individual strain parameters were used. For the parameter *C_f_*, a deterministic value determined for the strain IMPC2.1 was used. It was assumed that the white cabbage matrix has limited impact on the growth rate of *L. paracasei* species (*C_f_* close enough to 1) and that the value determined for IMPC2.1 would be applicable for the other tested strains. A deterministic *C_f_* value was already used in Le Marc et al. (2021b), providing satisfactory results for the stochastic modeling of mesophilic *B. cereus* in reconstitute infant formula.

For the parameter *N_max_*, the model developed for strain IMPC2.1, was used also applied in the simulations. Based on the experimental data in broth (plate count experiments) which did not exhibit significant differences between the *N_max_*-values observed between the four strains studied, the *N_max_* was assumed to be not strain-dependent, also in white cabbage experiments. To account for product variability, a normal distribution for the initial pH based on the minimum and standard deviation of pH measurements in cabbage, was used.

Simulations (10,000 iterations) were performed using an in-house program developed in the MATLAB language. These growth simulations were used to estimate the probability to reach the targeted probiotic level fixed at 7 log_10_ CFU/g as a function of time. The results were also compared to experimental data for strain IMPC2.1 in white cabbage (the growth data were either used for calculating the food correction factor and the maximum population density model) and with other fully independent data.

## Results

### Molecular Characterization

Strains were analyzed and clustered in a dendrogram using rep-PCR fingerprints (Supplementary Figure S1). The bacterial strains showed reproducible and specific patterns. The number of bands generated per strain by primers ranged between 12 and 22 for REP-1R-Dt/REP-2R-Dt, 11–18 for REP 1R-I/REP 2R-I, 20–25 for BOX A1R and 23–28 for (GTG)_5_. A cluster analysis of the complete set of rep-PCR data differentiated *L. paracasei* strains from the outgroup strain *L. plantarum* ITM21B. All *L. paracasei* strains were linked at a similarity coefficient level of 72.8%. In particular, P101 and P40 strains clustered together with a similarity level of 74.2%. Similarly, the strains IMPC2.1 and IMPC4.1 showed a cluster similarity of 86.1%. Therefore, IMPC2.1 and IMPC4.1 appeared to be closely related strains.

### Growth Model Parameters

**Figure 1:**
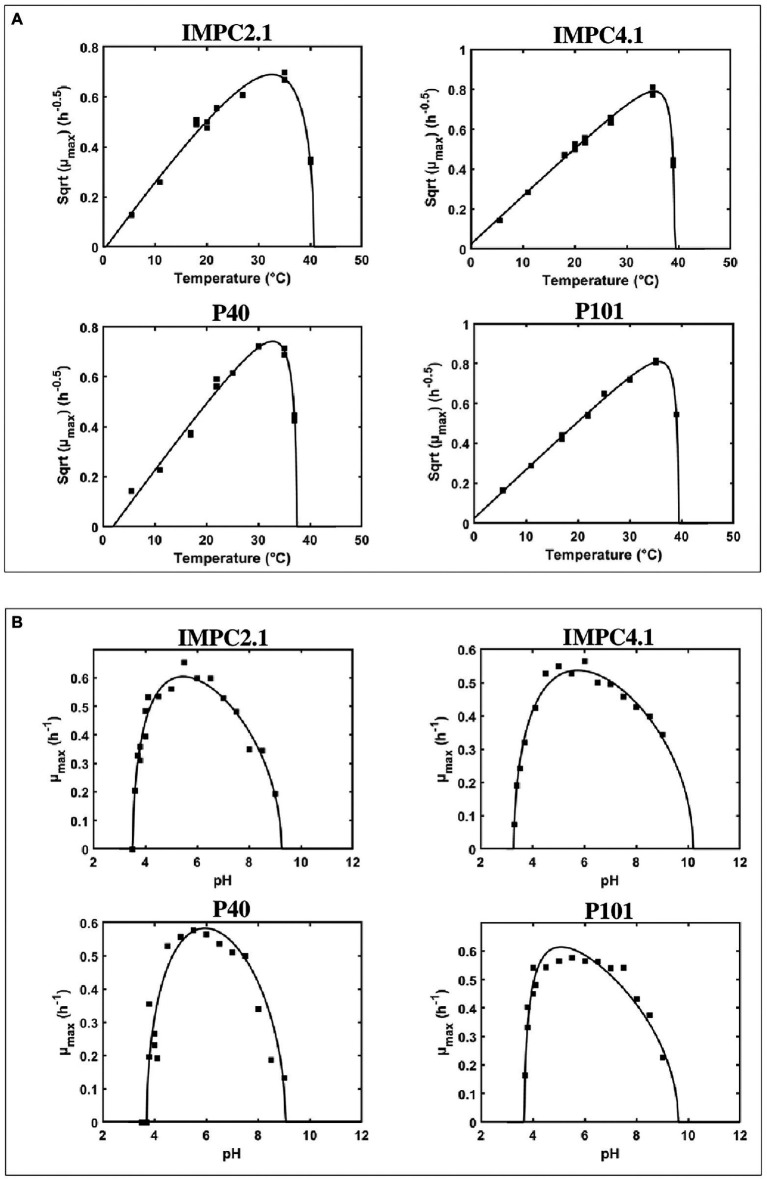
Effect of temperature **(A)** and of pH **(B)** on the maximum specific growth rate (*μ_max_*) of different strains of *Lacticaseibacillus paracasei*. Comparison between the fitted model and observed maximum specific growth rates (■).

Equation (1) was used to fit *μ_max_* data for each strain as a function of temperature (T) and pH giving the growth model parameters reported in Table 2. Growth of strains was tested at temperatures ranging from 5.5°C to 40°C and strain IMPC2.1, being the only strain able to grow at 40°C, showed a significantly higher *T_max_* value compared to the other strains. The minimal temperature cardinal values (*T_min_*) of two strains (IMPC4.1 and P101) resulted to be lower than 0°C highlighting a strain variability in growth abilities at low temperatures (Table 2; Figure 1A). However, this can be considered non-significant as the confidence intervals for the different strains overlap.

**Table 2:** Estimated growth cardinal parameters and 95% CI for the *Lacticaseibacillus paracasei* strains.

Parameters^a^	IMPC2.1	IMPC4.1	P40	P101
*μ_opt,MRS_* (h^−1^) for T	0.48 [0.43; 0.52]	0.62 [0.576; 0.672]	0.55 [0.50; 0.60]	0.65 [0.609; 0.699]
*T_min_* (°C)	0.61 [−2.11; 3.33]	−0.93 [−2.91; 1.05]	1.95[−0.41; 4.30]	−0.97 [−2.64; 0.71]
*T_opt_* (°C)	32.63 [31.24; 34.01]	35.31 [34.08; 36.54]	32.8 [31.78; 33.82]	35.67 [34.53; 36.80]
*T_max_* (°C)	40.74 [40.33; 41.15]	39.26 [39.06; 39.46]	37.46 [37.17; 37.75]	39.42 [39.08; 39.77]
Number of data for T	14	14	13	14
*pH_min_*	3.43 [3.37; 3.49]	3.23 [3.21; 3.25]	3.70 [3.69; 3.70]	3.50 [3.41; 3.59]
*pH_max_*	9.53 [9.18; 9.88]	10.44 [9.31; 11.58]	9.32 [8.68; 9.95]	9.69 [9.02; 10.35]
*Q*	0.29 [0.13; 0.45]	0.29 [0.07; 0.51]	0.27 [−0.09; 0.64]	0.28 [0.03;0.52]
Number of data for pH	17	16	16	16

a *μ_opt,MRS_:* *optimum maximum specific growth rate in modified MRS; *T_min_*, *T_opt_* and *T_max_* are the minimum, optimum and maximum temperature for growth; *pH_min_* and *pH_max_* are the minimum, maximum pH for growth, respectively, and *Q* is a shape parameter. Parameters were calculated as reported in section Temperature and pH Models*.

The evolution of *μ_max_* as a function of pH showed similar pattern for the four strains studied: *μ_max_* increased sharply from *pH_min_* to *ca.* pH 4.5, followed by more gradual decrease when pH increased from *ca.* pH 6 towards pH 9.1 (Figure 1B). All strains were able to grow at pH 9.1, while a variability was found regarding the capacity to grow at low pH values. In particular, no growth was observed at pH 3.7 for strain P40, pH 3.5 for IMPC2.1 and P101, while IMPC4.1 was able to grow even at pH 3.3. The minimum pH values (*pH_min_*) estimated for each *L. paracasei* strain ranged between pH 3.23 and pH 3.70, and the lowest value was observed for strain IMPC4.1. The estimates for *pH_max_* values were from 9.32 to 10.44, while the shape parameter *Q* was close to 0.3 for all the strains. As shown in Figure 1B, the fitted Equation (3) showed reasonable agreement with the observed *μ_max_* values (*R*^2^ = 0.94, RMSE = 0.027).

When considering the fitting for temperature and pH, it appears that a similar genetic pattern was not indicative of the same growth performance, and the results indicated some strain variability within the same species.

### Comparison Between “*in silico*” and “*in food*” Kinetics

**Figure 2:**
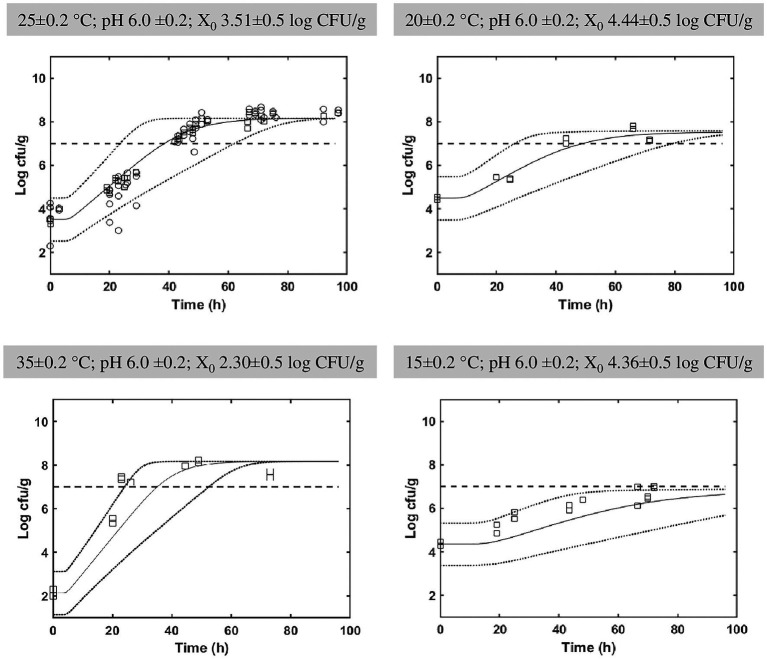
Comparison between in food growth of *Lacticaseibacillus paracasei* from all experimental data relevant to Table 1 (squares), additional data generated at 25°C not included in Sarvan et al. (Sarvan et al., 2013; circles) and the prediction *in silico* (line) with confidence interval (dashed lines). The stochastic simulations were performed using the normal distributions by the calculated mean values of the growth parameters and relevant standard deviations obtained for the four *Lacticaseibacillus paracasei* strains (Table 2).

The fermentation experiments in mild processed cabbage demonstrated the presence of strain *L. paracasei* IMPC2.1, which represented the totality of LAB isolates, and its growth on the vegetable matrix as ascertained by REP-PCR analysis performed at each sampling time (Figure 2). Kinetic parameters relevant to the growth of the strain were obtained fitting experimental data with the primary growth model (Rosso et al., 1996).

Supplementary Figure S2 shows the evolution of log_10_(*N_max_*) of IMPC2.1 strain in cabbage as a function of temperature. Note that at 15°C, the threshold concentration of 7 log_10_ CFU/g has not been reached.

Experiments performed *in food* suggested that a different growth ability of the strain IMPC2.1 in the aqueous (4% NaCl brine with water activity 0.993) and solid (cabbage having a lower a_w_, 0.989) phases occurred (data not shown); therefore, in the present study, the a_w_ parameter was not taken into account for the model development. However, to take into account the different growth ability of strains in broth and in a food matrix, the correction factor *C_f_*, was calculated. The correction factor *C_f_* (estimated as 0.85) and the parameter *K* (“work to be done” by the bacteria before growing) with others estimated parameters for simulations, are reported in Table 3.

**Table 3:** Kinetic parameters relevant to the growth of the *Lacticaseibacillus paracasei* IMPC2.1 strain in the mild processed cabbage for simulations.

Kinetic parameter^a^	
*C_f_*	0.85
*K*	1.77
*a_0_*	4.74
*a_1_*	0.14
*T_c_*	24.5

a *C_f_*: *correction factor accounting for the impact of food matrix on bacterial growth rate; *K*: lag time assumed to be independent on the experimental conditions but dependent on cell history and food matrix; *a_0_, a_1_* and *T_c_*: model parameters, where *T_c_* represents the temperature above which the *N_max_* was constant*.

To demonstrate the model applicability to fermentative process operated by the *L. paracasei* species, the stochastic simulations were performed using the normal distributions by the calculated mean values of the growth parameters and relevant standard deviations obtained for the four *L. paracasei* strains. *Lacticaseibacillus paracasei* growth simulations were performed, using a Monte Carlo stochastic approach, for different conditions with various intrinsic (pH), environmental (fermentation temperature) and inoculum load conditions and compared to experimental counts.

**Figure 3:**
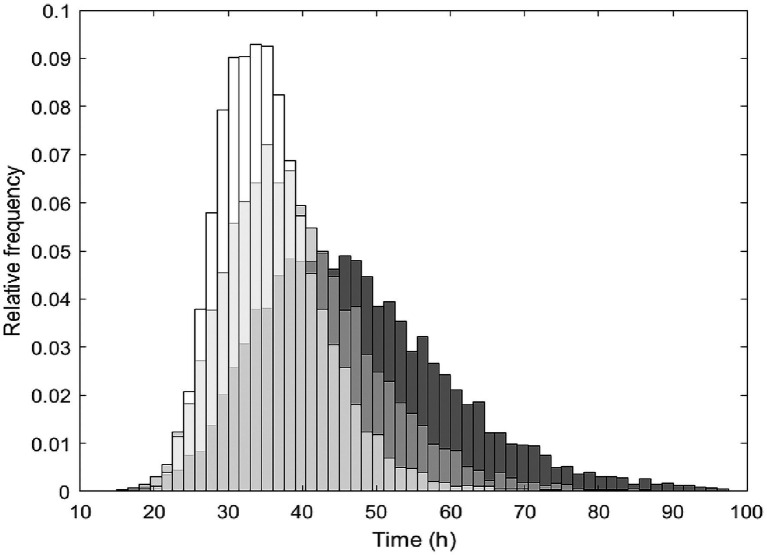
The simulated distributions of *Lacticaseibacillus paracasei* population randomly generated that reached the targeted probiotic level fixed at 7 log_10_ CFU/g during fermentation time at 20°C (grey), 25°C (light grey) and 35°C (white). Simulations were obtained in cabbage considering a virtual initial probiotic inoculum of 4 log_10_ CFU/g ± 0.5 at pH 6.0 ± 0.2 during 4 days.

Figure 2 shows the bacterial counts from experimental data relevant to Table 1 (squares), additional data appeared at 25°C and not included in Sarvan et al. (Sarvan et al., 2013; circles) and the prediction *in silico* (line) with confidence interval (dashed lines). The simulation results are consistent with the experimental observations. Discrepancy between prediction and observations only appears at 25°C and were relevant to the additional experimental data. The distribution of simulations leads to predict the fermentation time needed to reach the targeted probiotic level 7 log_10_ UFC/g for *L. paracasei* population at given temperatures (e.g., Figure 3).

### Prediction of Growth Probability for *Lacticaseibacillus paracasei* spp. in Cabbage

In order to predict the fermentation time and conditions allowing the growth of *L. paracasei* until reaching the targeted probiotic level fixed at 7 log_10_ CFU/g, the Monte Carlo simulation approach was used. As reported in Figure 3, the distributions of simulations show the reaching of the targeted probiotic level for *L. paracasei* population during fermentation time at 20°C (grey), 25°C (light grey) and 35°C (white).

The growth of *L. paracasei* in cabbage was simulated considering N_0_ 4.0 ± 0.5 and pH 6.0 ± 0.2 for all the simulations at the different temperatures. In these simulations, the mean of the log counts at *t* = 0 was used to allow a better comparison between temperature conditions. The distribution of simulation over the time at 15°C was not included since as shown in Supplementary Figure S2, *L. paracasei* population will not reach this threshold level after 96 h. As illustrated in Figure 3, simulation frequency distribution highlighted shorter time to reach 7 log_10_ CFU/g at 35°C than 25°C and 20°C. However, at 20°C the bacterial population was predicted to reach this threshold with a probability of 99.6%.

**Figure 4:**
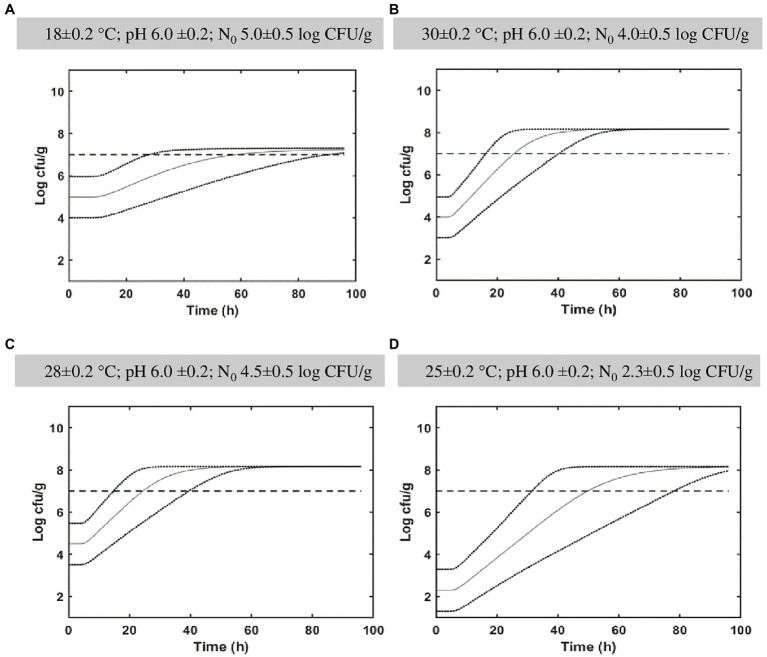
*In silico* growth simulations and probability of *Lacticaseibacillus paracasei* to reach the targeted probiotic level fixed at 7 log_10_ CFU/g in cabbage. Simulations were obtained in cabbage during 4 days at different conditions (**A**: 18°C, pH 6.0, N_0_ 5.0; **B**: 30°C, pH 6.0, N_0_ 4.0; **C**: 28°C, pH 6, N_0_ 4.5; **D**: 25°C, pH 6.0, N_0_ 2.30). The distribution of *Lacticaseibacillus paracasei* population is indicated together with a targeted probiotic level threshold fixed at 7 log_10_ CFU/g.

Figure 4 represents *in silico* growth simulations for different fermentation temperatures. The Monte Carlo simulations were performed to determine the *L. paracasei* spp. population distribution as a function of time and the associated probability percentage to overpass the targeted probiotic level of 7 log_10_ CFU/g at 96 h. For cabbage fermented at 18°C, simulations showed a probability of 98.2% (Figure 4A) at studied conditions (pH 6.0 ± 0.2, N_0_ 5.0 ± 0.5 log_10_ CFU/g) at the end of fermentation (96 h). At 25°C (pH 6.0 ± 0.2) simulation showed a probability of 99.7% at 96 h of fermentation notwithstanding the log counts at *t*_0_ = 2.3 ± 0.5 log_10_ CFU/g (Figure 4D). At the conditions reported in Figure 4B (30 ± 0.2°C; pH 6.0 ± 0.2; N_0_ 4.0 ± 0.5 log_10_ CFU/g) and Figure 4C (28 ± 0.2°C; pH 6.0 ± 0.2; N_0_ 4.5 ± 0.5 log_10_ CFU/g), the probability percentage to overpass the probiotic level was 100% at 96 h.

## Discussion

Classically, predictive microbiology has been mainly applied for assisting food industries in food safety hazard management with growth prediction of unwanted contamination occurring with unknown low initial contamination level of pathogenic or spoilage bacteria (e.g., Cassin et al., 1998; Oscar, 1998; Nauta, 2002; Valerio et al., 2015a). On the contrary, the application of predictive microbiology to pro-technological microorganisms considers an established high initial inoculum load of a well-characterized strain or mixtures to start the fermentation process. Moreover, to reach the beneficial effects and technological properties of the strain, predictive goal of modeling needs to include the maximum density parameter reached. These aspects should be taken into account in the modeling techniques and in particular dealing with LABs which possess the ability to grow at low pH and to survive gastrointestinal transit, thus considered probiotic candidates.

The four strains used in the current study have different origins: strains P40 and P101 were isolated from table olives, while strains IMPC4.1 and IMPC2.1 were isolated from human feces and characterized for probiotic features, with strain IMPC2.1 previously used to ferment vegetables (white cabbage, table olives, artichokes), fish or included in an edible coating for dried apple slices to obtain probiotic foods. In particular, strain IMPC2.1 was selected since its probiotic features were established in *in vivo* studies and its health benefits on constipated patients observed after supplementing their diet with ready-to-eat foods enriched with 9 log_10_ CFU of cells per portion, corresponding to about 7 log_10_ CFU/g (Valerio et al., 2010, 2015b; Riezzo et al., 2012). Therefore, the level of 7 log_10_ CFU/g was considered as targeted probiotic level in the current study.

The aim of the present work was, in the context of process optimization, to increase knowledge on the growth ability of *L. paracasei* spp. in different fermentation conditions, and to identify the fermentation conditions and the time needed to reach the targeted probiotic level (7 log_10_ CFU/g) in cabbage with mathematical model.

To pilot the fermentation of cabbage products, knowing the *L. paracasei* spp. growth ability, is of great interest since cabbage is cultivated all over the world due to its abundance and low price and is, as a fermented vegetable, one of the most popular and traditional products. German sauerkraut, Korean kimchi or pickled Chinese cabbage fermentations were widely studied both naturally and with starter cultures and often LABs were involved. The *L. paracasei* HD 1.7 was used as a starter to modulate the bacterial community and metabolome profile during fermentation of Chinese cabbage (Zhao et al., 2018) and showed a growth behavior of the *L. paracasei* strain at 17–20°C (Zhao et al., 2016) quite similar to that predicted with our model (Figure 4A; 18°C) indicating its potential to estimate the growth of *L. paracasei* species in fermented cabbage. Generally, the German sauerkraut contains low amount of bioactive molecules which are degraded during the long fermentation process and processing (Sarvan et al., 2013). As demonstrated by several authors, a strategy to preserve the bioactive molecules glucosinolates, consists in operating a mild fermentation process in a short time (Sarvan et al., 2013; Nugrahedi et al., 2015; Sun et al., 2021). A thermal treatment (blanching) can be performed before fermentation to inactivate the thermolabile endogenous myrosinase and obtain a higher glucosinolate content compared to traditionally fermented cabbage. As demonstrated in Sarvan et al. (2013), the effect of blanching (100°C for 5 min) followed by fermentation (4% brine at 25°C) with the probiotic strain *L. paracasei* IMPC2.1, allowed to partially preserve the GSs content in the final product.

In order to predict the bacterial behavior in a food matrix, the impact of intrinsic and extrinsic parameters on bacterial growth is estimated by the cardinal growth parameters for pH, temperature and water activity. Regarding the parameters for temperature, they were determined in broth by applying the classical CTMI model (Rosso et al., 1993) while, to describe the relationships between *μ_max_* and [H^+^] in the suboptimal range and the gradual decrease of *μ_max_* in the supraoptimal range, the equations based on a previous study (Presser, 2001), were proposed. Most of the pH models used in predictive microbiology, including the popular cardinal pH model, assume a close-to-linear evolution of the growth rate as a function of suboptimal pH. However, the *L. paracasei* data from this study show a different pattern, with *μ_max_* increasing almost linearly with the proton concentration [H^+^] near pH_min_. A similar pattern has also been observed for other microorganisms, including *L. plantarum* (Aryani et al., 2016), and *B. cereus sensu* lato (Le Marc et al., 2021a).

Fermentation processes and food matrix composition can have a serious impact on the growth rate of strains when conditions become stressful for them as represented by the biphasic state of food matrix. In fact, the microstructure of fermented cabbage affects the microbial growth (aqueous/brine and solid/vegetable). Actually, *L. paracasei* suspended in brine, where microbial cell mobility is not constrained, displayed a planktonic growth pattern as in synthetic medium (Wilson et al., 2002). Instead, other cells adhered to the cabbage surface and their growth pattern turns from planktonic to colonial. As reported by Skandamis and Jeanson (2015), bacteria on the surface of foods, such as meat and vegetables, grow as colonial, initially in two dimensions (mono-layer), whereas the center of colony gradually develops in the third dimension most likely upward. Depending on the nutrient content of a medium, the cells growth rate follows the order: planktonic ≥ submerged > surface (Wilson et al., 2002; Theys et al., 2008) and the lag times too, between these three different statuses of growth. In fact, results from the current study suggested that the inoculated strain grew more slowly on cabbage than in broth. For this reason, the a_w_ parameter was not taken into account for the model development and this aspect is actually under investigation to complete the growth model for this species, applied to a biphasic state food matrix.

To compare the ability of blanched white cabbage to support the growth of the studied strain in comparison with the growth obtained in culture medium, the food correction factor *C_f_* was introduced. The *μ_max pred_* value predicted in white cabbage was simply the predicted value in mMRS multiplied by this correction factor and was in good agreement with experimental observations (Bias factor *B_f_* = 0.97; Accuracy *A_f_* = 1.18).

As demonstrated by the fermentation experiments in blanched white cabbage (Table 1; Figure 2), the presence of *L. paracasei* IMPC2.1 was ascertained at each sampling time. The strain was able to growth on the vegetable matrix reaching the targeted probiotic level in different fermenting conditions also using a very low inoculation load (about 2 log_10_ CFU/g). The targeted level was even reached at temperature higher than 20°C.

The robustness of predictive model of *L. paracasei* spp. growth behavior is fundamental for its practical applicability in fermented cabbage. Therefore, the mean and standard deviation values of the growth cardinal parameters of all *L. paracasei* studied strains were used to perform the *in silico* growth simulations and to estimate the probability to reach and overpass the targeted probiotic level (7 log_10_ CFU/g) in cabbage. Results indicated that a storage temperature (30°C) close to the T_opt_ value of *L. paracasei* strains (ranging from 32.63°C to 35.67°C, Table 2) allowed to reduce time of food processes to about 24 h (Figure 4B). It should be considered that the optimal conditions for cabbage fermentation to maintain antioxidant properties include temperatures in the range 18–22°C using long time of fermentation (62 days; Montet et al., 2014; Berger et al., 2020). However, the long fermentations have a significant impact on the glucosinolate degradation as demonstrated by Palani et al. (2016). Therefore, the reduction of fermentation time could considerably improve the nutraceutical value of the final product. In the current study, simulations indicated a probability of 98.2% to reach the targeted probiotic level at 18°C using an inoculum of 5.0 ± 0.5 log_10_ CFU/g and pH 6.0 ± 0.2, after 96 h (Figure 4A). Simulations showed also that pH did not affect the fermentation time, probably because of the natural ability of LAB strains to grow in acidic environment, due to their own production of organic acids. Indeed, the impact of lactate and undissociated lactic acid on bacterial growth and the lactic acid production rate of LAB strains, has been studied (Venkatesh et al., 1993; Aghababaie et al., 2015) since the ability of LABs to grow in acidic environments makes LAB strains good candidates as biopreserving starter cultures for fermented foods (Laitila et al., 2004).

As a result, the determination of the growth behavior in broth of a representative number of strains led to the development of a robust predictive model applicable to real food processing scenario to select the most suitable conditions for cabbage fermentation. The growth cardinal parameters determined for these strains can be also exploited to simulate the fermentation process in other food matrices after performing the relevant challenge tests in food.

Further studies are required for completely characterize the impact of other environmental and intrinsic factors (a_w_, organic acids, physical structure, etc.) on *L. paracasei* growth.

## Conclusion

A growth model was developed to predict the effect of temperature and pH on the maximum specific growth rate of bacterial strains. In particular, in the current study the cardinal growth parameters of 4 *L. paracasei* strains selected for their technological and functional properties, were reported and discussed. The development of a predictive growth model for *L. paracasei* strains by the introduction of a new pH model will allow to select the fermentation conditions to reach the targeted probiotic level of 7 log_10_ CFU/g of fermented cabbage corresponding to food portions containing about 9 log_10_ CFU probiotic viable cells.

Further efforts will aim to characterize *L. paracasei* strains for water activity parameters, considering both NaCl and sugar effects, and for organic acid production in optimal and suboptimal growth conditions to take into account the effects on their own growth and the inhibitory effects against food spoilage or pathogen contaminants. The final aim is to increase the robustness of the model by generating a database of growth parameters of other *L. paracasei* strains (to account for strain variability) and generate challenge test data for these strains in other food matrices.

In conclusion, this study aims to widen the field of food microbiological research to the application of predictive microbiology to pro-technological microorganisms for *in silico* fermentation process optimization in an industrial context.

## Data Availability Statement

The raw data supporting the conclusions of this article will be made available by the authors, without undue reservation.

## Author Contributions

MDB and YLM: conceptualization, investigation, and writing—original draft. ARB, PDB, and SLL: investigation. PL and FP: writing—review and editing. FV: conceptualization, writing—review and editing, and supervision. All authors contributed to the article and approved the submitted version.

## Funding

This work was supported by Project PON ARS01_00783 ALIFUN—“Development of functional foods for the traditional Italian food products innovation” and Research agreement between CNR-ISPA and Roboqbo Srl.

## Conflict of Interest

The authors declare that the research was conducted in the absence of any commercial or financial relationships that could be construed as a potential conflict of interest.

## Publisher’s Note

All claims expressed in this article are solely those of the authors and do not necessarily represent those of their affiliated organizations, or those of the publisher, the editors and the reviewers. Any product that may be evaluated in this article, or claim that may be made by its manufacturer, is not guaranteed or endorsed by the publisher.

## Footnotes

^1^ www.symprevius.eu

^2^ www.ispa.cnr.it/Collection
