# Supplementary figures and images for: A Predictive Growth Model for Pro-technological and Probiotic Lacticaseibacillus paracasei Strains Fermenting White Cabbage
Source: Front Microbiol. 2022 Jun 6;13:907393. doi: 10.3389/fmicb.2022.907393 (PMC9207389; doi:10.3389/fmicb.2022.907393)

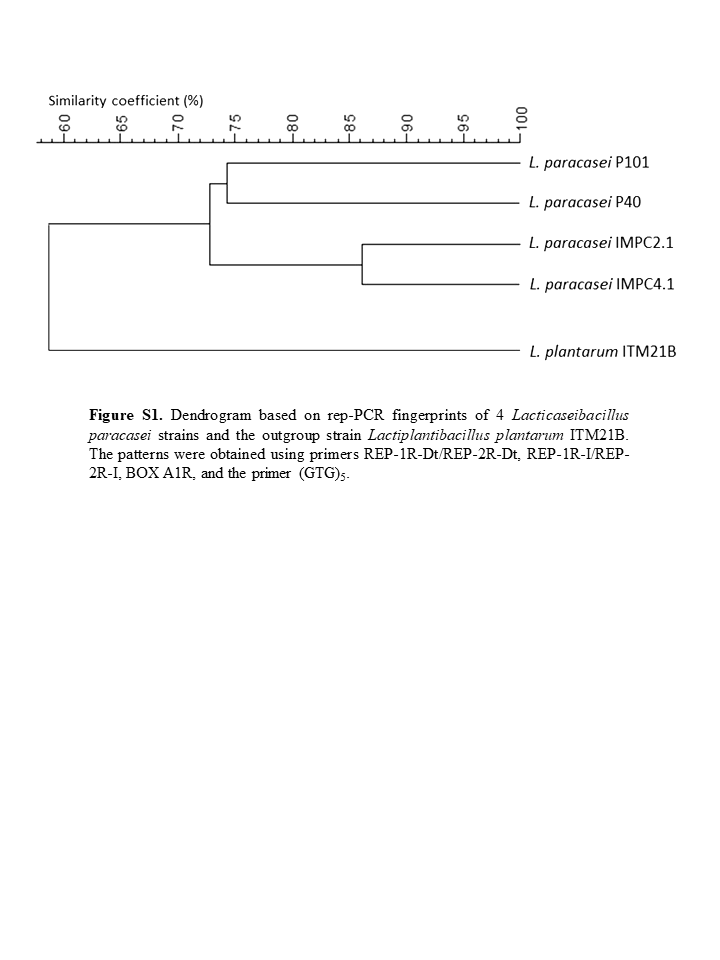

Supplement: Supplementary file 1 [file Image_1.tif]

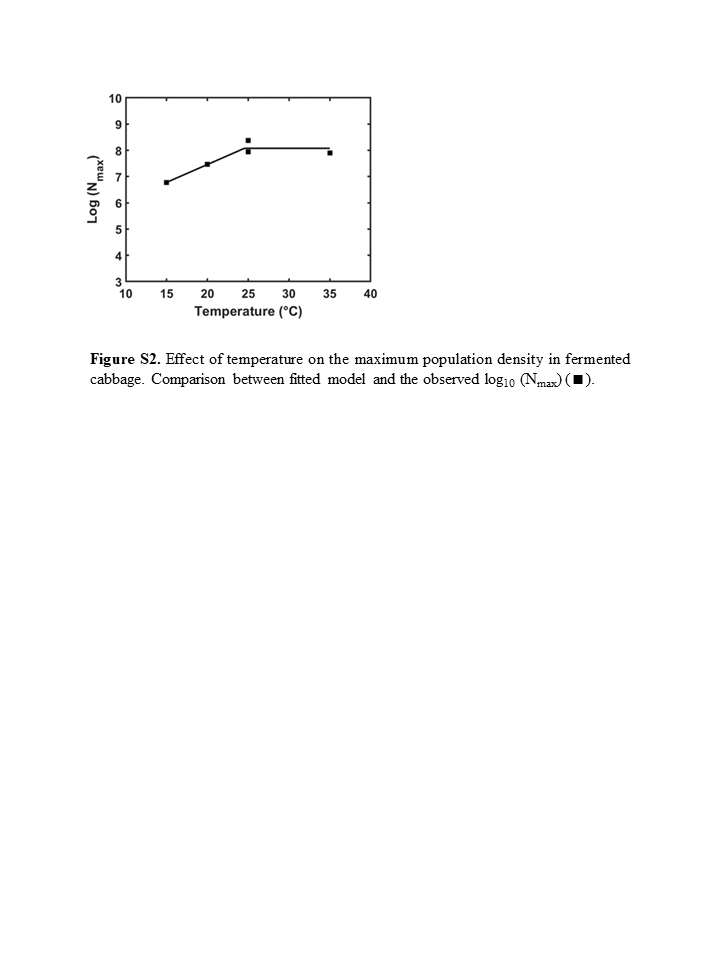

Supplement: Supplementary file 2 [file Image_2.tif]
